# Combinatorial Biosynthesis of Sulfated Benzenediol Lactones with a Phenolic Sulfotransferase from Fusarium graminearum PH-1

**DOI:** 10.1128/mSphere.00949-20

**Published:** 2020-11-25

**Authors:** Linan Xie, Dongliang Xiao, Xiaojing Wang, Chen Wang, Jing Bai, Qun Yue, Haitao Yue, Ye Li, István Molnár, Yuquan Xu, Liwen Zhang

**Affiliations:** a Biotechnology Research Institute, The Chinese Academy of Agricultural Sciences, Beijing, People’s Republic of China; b Southwest Center for Natural Products Research, University of Arizona, Tucson, Arizona, USA; c Microbial Pharmacology Laboratory, Shanghai University of Medicine and Health Sciences, Shanghai, People’s Republic of China; d School of Chemistry, Biology and Material Engineering, Suzhou University of Science and Technology, Suzhou City, Jiangsu Province, People’s Republic of China; e Department of Biology and Biotechnology, Xinjiang University, Urumqi, People’s Republic of China; f National Engineering Lab for Cereal Fermentation Technology, Jiangnan University, Wuxi, People’s Republic of China; University of Georgia

**Keywords:** *Fusarium*, combinatorial biosynthesis, phenolic sulfotransferase

## Abstract

Total biosynthesis or whole-cell biocatalytic production of sulfated small molecules relies on the discovery and implementation of appropriate sulfotransferase enzymes. Although fungi are prominent biocatalysts and have been used to sulfate drug-like phenolics, no gene encoding a sulfotransferase enzyme has been functionally characterized from these organisms. Here, we identify a phenolic sulfotransferase, FgSULT1, by genome mining from the plant-pathogenic fungus Fusarium graminearum PH-1. We expressed FgSULT1 in a Saccharomyces cerevisiae chassis to modify a broad range of benzenediol lactones and their nonmacrocyclic congeners, together with an anthraquinone, with the resulting unnatural natural product (uNP) sulfates displaying increased solubility. FgSULT1 shares low similarity with known animal and plant sulfotransferases. Instead, it forms a sulfotransferase family with putative bacterial and fungal enzymes for phase II detoxification of xenobiotics and allelochemicals. Among fungi, putative FgSULT1 homologues are encoded in the genomes of *Fusarium* spp. and a few other genera in nonsyntenic regions, some of which may be related to catabolic sulfur recycling. Computational structure modeling combined with site-directed mutagenesis revealed that FgSULT1 retains the key catalytic residues and the typical fold of characterized animal and plant sulfotransferases. Our work opens the way for the discovery of hitherto unknown fungal sulfotransferases and provides a synthetic biological and enzymatic platform that can be adapted to produce bioactive sulfates, together with sulfate ester standards and probes for masked mycotoxins, precarcinogenic toxins, and xenobiotics.

**IMPORTANCE** Sulfation is an expedient strategy to increase the solubility, bioavailability, and bioactivity of nutraceuticals and clinically important drugs. However, chemical or biological synthesis of sulfoconjugates is challenging. Genome mining, heterologous expression, homology structural modeling, and site-directed mutagenesis identified FgSULT1 of Fusarium graminearum PH-1 as a cytosolic sulfotransferase with the typical fold and active site architecture of characterized animal and plant sulfotransferases, despite low sequence similarity. FgSULT1 homologues are sparse in fungi but form a distinct clade with bacterial sulfotransferases. This study extends the functionally characterized sulfotransferase superfamily to the kingdom Fungi and demonstrates total biosynthetic and biocatalytic synthetic biological platforms to produce unnatural natural product (uNP) sulfoconjugates. Such uNP sulfates may be utilized for drug discovery in human and veterinary medicine and crop protection. Our synthetic biological methods may also be adapted to generate masked mycotoxin standards for food safety and environmental monitoring applications and to expose precarcinogenic xenobiotics.

## Abstract (precis)

Sulfation is an expedient strategy to increase the solubility, bioavailability, and bioactivity of nutraceuticals and clinically important drugs. However, chemical or biological synthesis of sulfoconjugates is challenging.

## INTRODUCTION

Sulfotransferases (SULTs) have been extensively studied as important enzymes for the metabolism of xenobiotics and drugs and for the modulation of endobiotics (hormones, bioamines, carbohydrates, and proteins) in humans and other organisms. However, much less attention was paid to their application in synthetic biology despite the importance of sulfate esters in drug development. Compared to the parent molecules, sulfated derivatives show better water solubility (1–3) and may display improved tissue distribution, including traversing the blood-brain barrier (4, 5). In some cases, sulfate esters exhibit reduced biological activities, as they are more accessible to membrane transporters, leading to increased efflux from cells and faster elimination from organs (2). In other cases, sulfated small molecules may function as prodrugs that are desulfated in target tissues, thereby releasing the active parent compound. Bioactivation of such prodrugs may be exploited for drug delivery, but this phenomenon also leads to often unrecognized health risks, such as in the case of masked mycotoxins (i.e., sulfoconjugates of fungal natural products that attain toxicity when desulfated in tissues) (6, 7). Importantly, there are also many small molecules that, when sulfated, retain or even gain biological activities (2). This may pose dangers as with precarcinogenic xenobiotics, where sulfation yields more reactive derivatives that damage proteins and DNA. At the same time, sulfation is a successful modification often employed by medicinal chemistry for various clinically important drugs, nutraceuticals, and food supplements. Prominent examples are the critically important antibiotics colistin sulfate and gentamicin sulfate, the antitumor drug sulfomercaprine, the anticoagulant heparin, the antitussive (cough suppressant) dibunate, and the antiosteoarthritis drug and food supplement chondroitin sulfate (8–11). Remarkably, sulfation increases the water solubility of the important antifungal drug micafungin 10-fold and dramatically enhances its bioactivity (12). Similarly, most of the pharmacologically important effects of the successful hair growth-promoting drug minoxidil is dependent on bioactivation by sulfation in the human body (13).

Chemical sulfation of alcohol or amine functional groups of complex bioactive molecules routinely involves expensive protection-deprotection steps that may employ hazardous or environmentally problematic reagents. Biological sulfation alleviates these problems by employing regio- and stereoselective SULT enzymes with an appropriate balance of substrate specificity and promiscuity. SULTs may be utilized as purified enzymes *in vitro*, although the required sulfo group donor cosubstrate 3′-phosphoadenosine 5′-phosphosulfate (PAPS) is expensive, and its *in situ* regeneration is challenging (14, 15). More practically, SULTs may be implemented in a whole-cell format for the total biosynthesis of the sulfated product or, most frequently, for the biocatalytic derivatizaton of a preformed substrate scaffold. For example, the production of chondroitin sulfate A and C was achieved by a two-step biocatalytic strategy using three sulfotransferases (10), while xeno- or endobiotic sulfoconjugates were obtained on a gram scale using a Saccharomyces cerevisiae expression system with human SULTs (16). The continued development of practical and economical biological sulfation methods requires the identification and characterization of appropriate SULT enzymes. However, SULTs have primarily been characterized from animals and plants, reflecting the important roles that these enzymes play in drug metabolism and xenobiotic transformations (1, 2, 17). These studies revealed two classes of SULTs: cytosolic enzymes that sulfate most xenobiotics and endobiotics in animals and plants, and the membrane-associated SULTs that modulate cellular signaling and molecular recognition by sulfating macromolecules such as carbohydrates and proteins in eukaryotes (18). In addition, a few selected bacterial SULTs have also been described, focusing on their contributions to the biosynthesis of sulfated natural products (19, 20). These studies also led to the discovery of the bacterial arylsulfate SULTs that catalyze sulfuryl transfer between phenolic small molecules without the involvement of PAPS. However, these arylsufate SULTs bear negligible structural resemblance to the PAPS-dependent cytosolic SULTs (21). Although reports have shown the ability of filamentous fungi to sulfate various compounds (8, 22), to the best of our knowledge, no curated fungal SULT has ever been deposited to the UniprotKB database, and no gene has been conclusively linked to a functionally characterized SULT in the kingdom Fungi.

Benzenediol lactones (BDLs) are drug-like polyketide natural products from fungi with wide-ranging bioactivities (23–27). BDLs are defined by a macrocyclic lactone ring fused to a 1,3-benzenediol moiety, with the connectivity of the benzene ring differentiating the two main BDL subgroups, resorcylic acid lactones (RALs, C2-C7 bond) and dihydroxyphenylacetic acid lactones (DALs, C3-C8 bond). The size of the macrolactone ring is another important characteristic of BDLs. Among RALs, radicicol with a 14-membered macrocycle (RAL_14_) displays cancer cell antiproliferative and heat shock response modulatory activities, while the RAL_12_ lasiodiplodin displays mineralocorticoid receptor antagonist and prostaglandin biosynthesis inhibitory activities (27, 28). The DAL_12_ compound 10,11-dehydrocurvularin modulates heat shock response and the immune system by inhibiting the p97 segregase (29, 30). We have been developing combinatorial synthetic biological methods using an engineered Saccharomyces cerevisiae chassis to extend the chemical space accessible to BDL biosynthesis toward unnatural natural products (uNPs, e.g., novel NP scaffolds and derivatives produced by recombinant biosynthetic pathways in domesticated host organisms). Thus, we established polyketide synthase (PKS) domain and subunit shuffling as practical methods to obtain uNP BDL scaffolds (23, 24, 31). We also implemented orthogonal “tailoring” enzymes, such as polyketide *O-*methyltransferases with edited regiospecificity, and a xenobiotic glucosyltransferase-methyltransferase detoxification module to produce uNPs by total biosynthesis or biotransformation (32, 33). However, no BDL sulfates are known to be produced as genuine *de novo* natural products. Moreover, only a single BDL (zearalenone) was shown to be sulfated during the course of phase II (conjugative) detoxification by plants and fungi (6, 22), but no other BDL congeners have ever been investigated as substrates for sulfation reactions. Thus, we were interested to demonstrate that combinatorial synthetic biology may be applied to recruit sulfation as another orthogonal tailoring step for uNP BDL biosynthesis.

The current study uses genome mining to identify FgSULT1 from Fusarium graminearum PH-1 as a candidate cytosolic PAPS-dependent SULT for the biotransformation of phenolic natural products (34, 35). We validate FgSULT1 as a versatile biocatalyst and use homology structural modeling and site-directed mutagenesis to show that FgSULT1 retains the typical fold and the active site architecture of characterized animal and plant SULTs. We also reveal that FgSULT1 homologues are not widely present in fungi but form a distinct clade with bacterial SULTs. This work provides the first functionally characterized SULT from the kingdom Fungi, and demonstrates total biosynthetic and biocatalytic synthetic biological platforms that can be adapted for the biosynthetic production of uNP sulfoconjugates for drug discovery and for the generation of standards for food safety and environmental monitoring applications.

## RESULTS

### Sulfation of lasilarin by Fusarium graminearum PH-1.

A pilot screening campaign of an in-house library of 49 filamentous Ascomycete fungi (Table S1 in reference 36) revealed that Fusarium graminearum PH-1 (CBS 123657) is able to biotransform the model BDL substrate lasilarin 1 to the more polar products 1a and 1b (Fig. S1 in reference 36). The mass-to-charge ratio (*m/z*) of the [M-H]^–^ ions of products 1a and 1b were both 80 atomic mass units (amu) higher than that of lasilarin, indicating that these products are sulfated derivatives (*m/z*, 399.1119, calculated 399.1113 for the product 1a parent ion in high-resolution mass spectrometry/mass spectrometry [HRMS/MS] with a mass error of 1.50 ppm; *m/z*, 399.1077 for product 1b with a mass error of 9.02 ppm). To validate these presumed sulfoconjugates, we used high-performance liquid chromatography (HPLC)-HRMS/MS to verify that the parent ions of products 1a and 1b give rise to daughter ions of 319 amu (the *m/z* of the pseudomolecular ion of lasilarin 1 in the negative mode [M-H]^–^) and 275 amu (the *m/z* of the most abundant daughter ion of lasilarin 1 when a collision energy of 20 eV is used in the negative mode; Fig. S1 in reference 36). Searching the extracted ion chromatograms of fermentation extracts for appropriate parent and daughter ions is a validated method to detect the presumed sulfoconjugates of flavonoids and other phenolic substrates (37).

### Identification of the sulfotransferase of Fusarium graminearum PH-1.

Considering that F. graminearum PH-1 is a notorious plant pathogen, its utility in the biotechnology industry for biotransformation is limited by regulatory concerns. Thus, we set out to identify the enzyme responsible for BDL sulfation in this strain to design a synthetic biological platform for phenolic small-molecule derivatization. Since no functionally characterized fungal sulfotransferase (SULT) was available in the data banks, we used the amino acid sequence of the human phenolic sulfotransferase 1A1 (SULT1A1, GenBank protein accession number NP_001046) as a bait to query the predicted proteome of F. graminearum PH-1 (35), considering that most phenolic small molecules are sulfated by the SULT1A subfamily members in animals (1, 2, 38). The search (e < 0.1) returned only one of the four putative F. graminearum enzymes (FGSG_02887, FGSG_02116, FGSG_05047, and FGSG_05481) that had been annotated to feature a sulfotransferase conserved domain each (Sulfotransfer_1, PF00685.27). This enzyme, designated FgSULT1, shares 26% identity over 81% coverage with human SULT1A1 and is expressed under culture conditions conducive to lasilarin 1 sulfation, as shown by reverse transcription-PCR of the mRNA derived from gene FGSG_02887 (*fgsult1*) (Fig. S2 in reference 36).

**FIG 1:**
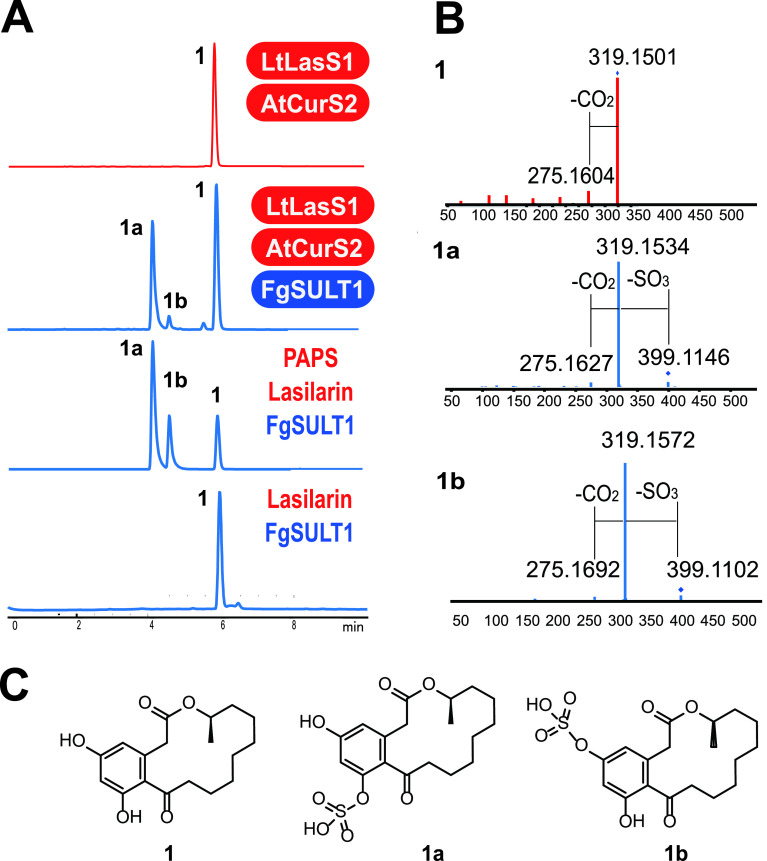
FgSULT1 is responsible for the sulfation of lasilarin 1. (A) Product profiles (reversed-phase HPLC-HRESIMS traces recorded as total ion chromatograms) of S. cerevisiae BJ5464-NpgA (66) expressing the indicated PKSs and FgSULT1 from F. graminearum PH-1 (upper two traces) or *in vitro* biocatalytic transformation of lasilarin 1 by the purified recombinant FgSULT1 enzyme with or without the sulfo group donor, 3′-phosphoadenosine 5′-phosphosulfate (PAPS) (lower two traces). (B) HRESIMS/MS spectra of lasilarin 1 and its sulfate esters 1a and 1b. (C) Structures of lasilarin 1 and its sulfate esters 1a and 1b.

Next, we expressed the intron-free *fgsult1* open reading frame in Saccharomyces cerevisiae BJ5464-NpgA, a host well suited to produce fungal enzymes and to reconstitute fungal polyketide biosynthetic pathways (32, 33). Lasilarin 1 was selected as the model substrate for sulfation. To avoid any potential cell permeability issues with an externally supplied substrate, we chose to produce lasilarin *in situ* in the recombinant yeast strain by coexpressing FgSULT1 with the highly reducing PKS-nonreducing PKS pair LtLasS1-AtCurS2 (24). The yeast strain produced lasilarin 1, together with compound 1a as the major sulfated product and compound 1b as the minor one, both with increased polarity compared to lasilarin 1 (Fig. 1A). The negative mode HRESIMS spectra of compounds 1a and 1b displayed [M-H]^−^ ions at *m/z* 399.1146 and 399.1102, respectively, both corresponding to the molecular formula C_18_H_23_O_8_S, consistent with lasilarin sulfate ester.

### *In vitro* reconstitution of the FgSULT1 reaction and structure elucidation of lasilarin sulfates.

The intron-free gene encoding FgSULT1 was cloned into the pACYCDuet-1 vector and expressed in E. coli Arctic Express (DE3) RIL. The His-tagged recombinant FgSULT1 protein was purified to homogeneity using Ni^2+^-nitrilotriacetic acid (NTA) affinity chromatography, and the reaction was reconstituted *in vitro* with lasilarin as the substrate and PAPS as the sulfo group donor cosubstrate. As shown in Fig. 1A, FgSULT1 successfully transformed lasilarin into its presumed sulfate esters 1a and 1b. No sulfated products were detected in the absence of PAPS. The improved production of compound 1b relative to 1a in the reconstituted reaction may indicate product inhibition with compound 1a. Alternatively, compound 1b may be degraded *in vivo* by other enzymes of the host during biotransformation.

The two apparent lasilarin sulfates 1a and 1b were isolated from large-scale *in vitro* reactions. Compound 1a was obtained in a sufficient amount (0.8 mg, isolated yield) for structure elucidation using nuclear magnetic resonance spectroscopy (NMR). The ^1^H NMR spectrum of compound 1a closely resembled that of lasilarin (24), but considerable chemical shift alterations were observed for the aromatic protons (H-4, δ_H_ 7.20, Δδ +0.86; H-6, δ_H_ 6.48, Δδ +0.14) (Table S2 in reference 36). The ^13^C NMR spectrum of compound 1a was also almost identical to that of lasilarin 1, except for a downfield shift for C-7 by 6.1 ppm (Table S2 in reference 36), indicating that sulfation took place at the 7-OH (39, 40). This was further confirmed by the presence of the 5-OH signal at *δ*_H_ 8.59 and the absence of the 7-OH signal in the ^1^H NMR spectrum of compound 1a. The yield of compound 1b (less than 0.3 mg) was too low for NMR characterization despite our best efforts. Nevertheless, the structure of compound 1b could still be elucidated as lasilarin 5-*O*-sulfate, considering that (i) The high resolution electrospray ionization mass spectrometry (HRESIMS)/MS profile of compound 1b is perfectly consistent with that of compound 1a, indicating that compounds 1a and 1b are lasilarin-*O-*sulfate regioisomers, and (ii) lasilarin has only two positions that can be sulfated: 7-OH, which is modified in compound 1a, and 5-OH, which consequently has to be the sulfation site in compound 1b. Lasilarin sulfates 1a and 1b are both new to nature.

### Combinatorial biosynthesis of BDL sulfates and biotransformation of anthraquinones.

**FIG 2:**
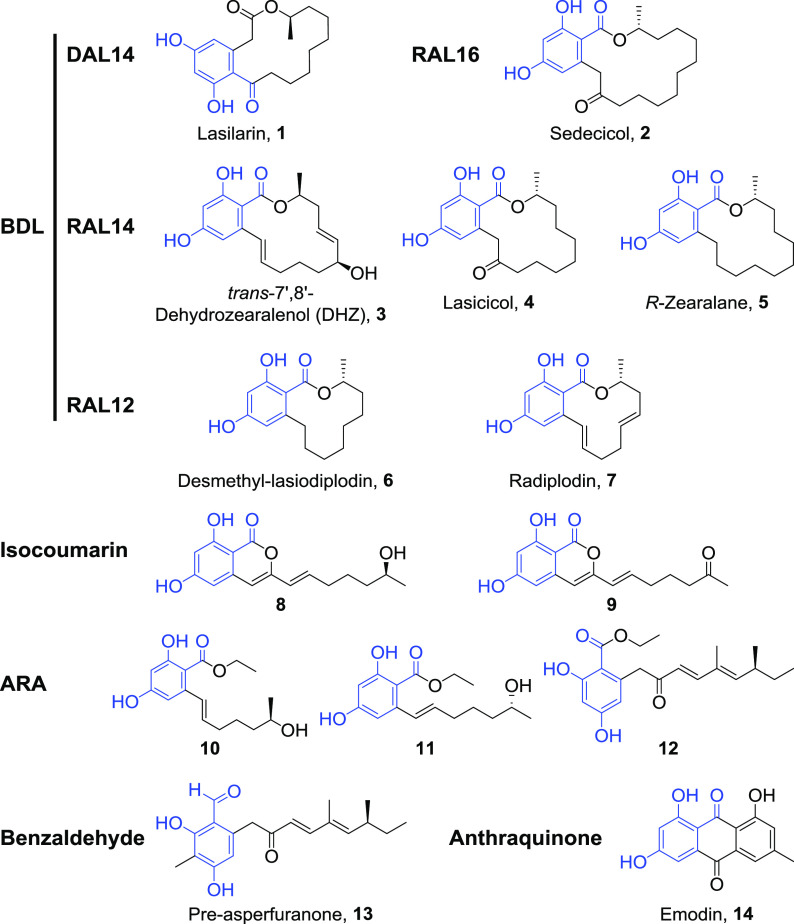
Structures of BDL and anthraquinone congeners that are sulfated by FgSULT1. The 2,4-dihydroxybenzaldehyde motif shared by these compounds is highlighted in blue. Table S8 in reference 36 lists the PKS pairs whose expression in yeast affords compounds 1 to 13. Fig. S3 in reference 36 shows additional model substrates investigated, while Table S3 and Fig. S4 in reference 36 provide detailed information on the HPLC-HRESIMS/MS identification of the detected products.

To investigate the substrate range of FgSULT1, we first assembled a collection of model substrates that represent the natural and “unnatural” BDL structure space (Fig. 2; Fig. S3 in reference 36). This included DAL_14_, DAL_12_, RAL_16_, RAL_14_, and RAL_12_ compounds, and nonmacrocyclic BDL congeners such as isocoumarins, acyl-resorcylic acid (ARA) ethyl esters, acyl-dihydroxyphenylacetic acid (ADA) ethyl esters, and an acyl benzaldehyde (Fig. 2; Fig. S3 in reference 36) (23, 24, 26, 32, 41). With the exception of zearalenol (22), none of these BDL congeners have been investigated as substrates for sulfotransferases. These 26 BDL congener scaffolds were all produced *in situ* by coexpressing the relevant PKS pairs with FgSULT1 in the S. cerevisiae chassis. Additional drug-like phenolic compounds such as flavonoids, anthraquinones, a stilbene, and a diarylheptanoid (Fig. S3 in reference 36) were also tested in a biocatalytic format by feeding them to an FgSULT1-expressing S. cerevisiae strain. We also tried feeding a range of simple phenols (Fig. S3 in reference 36), the widely used SULT model substrates 7-hydroxycoumarin 42 and *p*-nitrophenol 43 (42–44), and another BDL congener, zearalenone 49. Fourteen of the 49 model substrates were successfully conjugated by FgSULT1, as confirmed by HRESIMS/MS analysis (Fig. 2; Fig. S4 in reference 36, and Tables S3 and S8 in reference 36). The accepted substrates include a wide range of BDL congeners such as macrocyclic RALs and DALs of various ring sizes, isocoumarins, ARA ethyl esters, a benzaldehyde, and an anthraquinone. This indicates that the size of the macrocycle, the geometry of the benzenediol lactone (RAL versus DAL), and even the presence of the macrocyclic ring itself is not an absolute requirement for substrate recognition. In contrast, only emodin 14 was transformed among the anthraquinones, and the enzyme appears to be reluctant to sulfate ADA, flavonoid, stilbene, diarylheptanoid, and steroid scaffolds. Similarly, simple phenols are not accepted as substrates either (Fig. S3 in reference 36). These results suggest that the presence of a 2,4-dihydroxybenzaldehyde motif that is common to all successfully conjugated scaffolds (Fig. 2) is necessary but not sufficient for FgSULT1 substrate turnover (cf. compounds 1 to 14 with BDL congeners 15 to 27 and flavonoids 28 and 29). This motif must be part of a more complex scaffold, since phenols 44 and 45 were not modified either. Importantly, the “universal” SULT substrates 7-hydroxycoumarin 42 and *p-*nitrophenol 43, neither of which feature the 2,4-dihydroxybenzaldehyde motif, were also not transformed, indicating that FgSULT1 has very different substrate requirements compared to most well-characterized animal and plant SULTs (2, 3, 42).

Compared to their corresponding unconjugated scaffolds, the lipophilicity of the sulfate esters decreased substantially, as shown by the large increase of their polarities during reversed-phase chromatography, and by the remarkable decrease of their calculated ClogP values (fragment-based calculation of the logarithm of the partition coefficient between *n*-octanol and water; Table S3 in reference 36; 45). While the “drug-likeness” of oral drug candidates is influenced by many factors, reduced ClogP values (in the range of 2.5 to 3.0) correlate with higher success rates in market introduction, due to more favorable bioavailability, pharmacokinetics, and (in some cases) drug potency and toxicity profiles (46).

### FgSULT1 belongs to a SULT family populated by bacterial and fungal enzymes.

A blastp search with FgSULT1 against the GenBank and Mycocosm deduced protein databases (accessed on 11 August and 15 August 2020, respectively) showed that homologues of this enzyme have a very sporadic phylogenetic distribution in the kingdom Fungi. Thus, homologues that belong to the same SULT subfamily (identity, >65%; coverage, >95%) exist only in *Fusarium* spp. (Ascomycota, Sordariomycetes, Hypocreales). A modest number of more distant fungal homologues were also found to be members of the same SULT family as FgSULT1 (identity, >45%; coverage, >90%). Most of these putative enzymes are encoded in the genomes of *Microdochium*, *Astrocystis*, and *Xylaria* spp. that belong to a different order (Xylariales) of the Sordariomycetes class than the Fusaria. One additional homologous family member was also found in Stanjemonium grisellum and Acremonium strictum (Sordariomycetes, Hypocreales) and in Microascus trigonosporus (Sordariomycetes, Microascales). Several putative enzymes belonging to the FgSULT1 family were also detected in two genera (*Hortaea* and *Aureobasidium*) that belong to the Dothideomycetes, a different class of the Ascomycota (Table S4 in reference 36).

**FIG 3:**
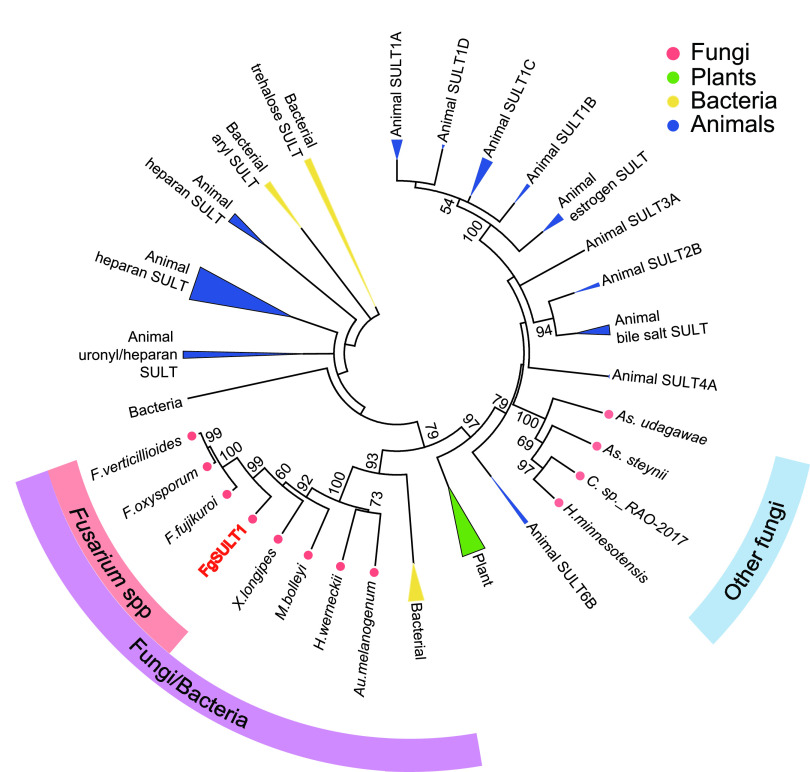
Phylogenetic analysis of FgSULT1. Phylogenetic tree of representative SULTs from animals (Homo sapiens, Mus musculus, Rattus norvegicus, and Danio rerio), a plant (Arabidopsis thaliana), bacteria, and fungi reconstructed using the maximum likelihood method. The *Fusarium* spp. subfamily of predicted SULTs (>65% identity to FgSULT1; >95% coverage) and the Fungi/Bacteria SULT family (>45% identity to FgSULT1; >90% coverage) are indicated by salmon and purple arcs, respectively. Other sulfotransferase domain-containing hypothetical proteins from fungi (23 to 27% identity to FgSULT1, labeled with a blue arc) form a sister clade to animal SULTs. The origins of the enzymes are color-coded as indicated. Numbers on branches show the percentage bootstrap support (when >50%) for each branch point, based on 1,000 pseudoreplicates. The log-likelihood of the phylogenetic tree is −12781.27. The substitution model used the Jones-Taylor-Thornton (JTT) model with uniform rates.

Instead of additional fungal homologues, the SULT family that FgSULT1 belongs to is typified by a large variety of enzymes from Proteobacteria and Cyanobacteria that feature the Sulfotransfer_1 domain (PF00685.27; Table S5 in reference 36). To better understand the evolutionary relationships of FgSULT1, a phylogenetic tree was constructed using the maximum likelihood method with 121 annotated SULTs deposited in the UniProtKB database from animals, plants, and bacteria, along with three representative FgSULT1 homologues from Fusaria, four additional fungal hypothetical proteins that belong to the same SULT family as FgSULT1, and four putative fungal sulfotransferase domain-containing proteins with distant similarities to FgSULT1 (Fig. 3A; Table S6 in reference 36). As expected, the clade housing members of the FgSULT1 family is made up by enzymes from *Fusarium*, *Xylaria*, *Michrodochium*, *Hortea* and *Aureobasidium* spp., together with bacterial homologues. This clade is basal to characterized SULTs from animals and plants, including those that conjugate xenobiotics and phenolic small molecules (e.g., the animal SULT1 family and the plant SOT family). The four sulfotransferase domain-containing hypothetical fungal proteins that display low similarities (23 to 27% identities) to FgSULT1 form a different clade, which is nestled among the animal SULT clades.

### Synteny analysis of loci encoding *fgsult1* homologues in fungi.

The *fgsult1* gene of F. graminearum PH-1 is located in a locus that may be related to sulfur recycling through the catabolism of the amino acids cysteine and methionine, as judged by the clustering of sulfite oxidase and molybdenum cofactor biosynthesis-related genes with *fgsult1* (Fig. S5 in reference 36). In Hortea werneckii, the *fgsult1* homologue (D0864_01954) clusters with a gene (D0864_01955) encoding a major facilitator superfamily transporter similar to the hydroxamate and catechol-type siderophore uptake transporters MirB in *Fusaria* (identity, >50%, coverage, >85%; Fig. S5 in reference 36, 47, 48). However, there is very limited to no apparent synteny in the genomes of *Fusarium*, *Xylaria*, *Hortea*, *Microdochium*, and *Aureobasidium* spp. in the loci where the *fgsult1* homologue resides. Importantly, the overwhelming majority of these fungal genomes do not contain obvious secondary metabolite biosynthetic gene clusters near *fgsult1* or its homologues.

### FgSULT1 displays the typical SULT architecture.

**FIG 4:**
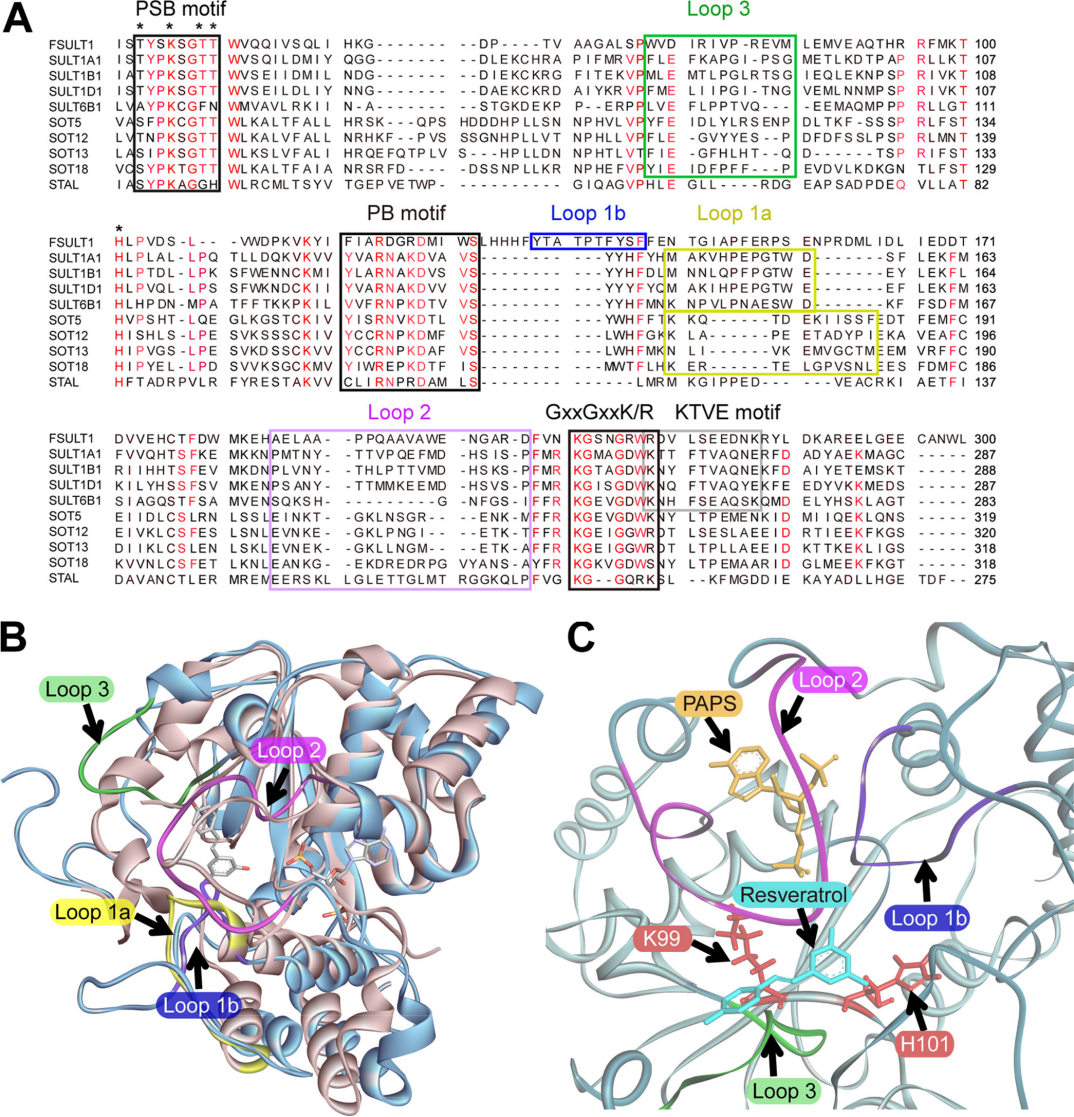
Sequence analysis and homology structure modeling of FgSULT1. (A) Sequence alignment of FgSULT1 with characterized SULTs from the mammalian SULT1 family, the plant SOT5, SOT12, SOT13, and SOT18 enzymes, and the prokaryotic SULT StaL. Black boxes show PAPS binding motifs, the gray box indicates the KTVE dimerization motif in animal SULTs, and additional colored boxes show the loop regions that gate the binding pocket in different SULTs (loops 1a, 1b, 2, and 3 as yellow, blue, purple and green boxes, respectively). Residues in the catalytic center that form hydrogen bonds with PAPS or the substrate are indicated with stars. FgSULT1 amino acid numbering is shown on the right. Amino acids conserved in >70% of the selected proteins are marked in red. (B) Structure superimposition of FgSULT1 (cartoon in blue) with the human cytosolic sulfotransferase SULT1B1 (3CKL, cartoon in sandy brown), including the substrates resveratrol and PAPS (shown as sticks). (C) Close-up of the substrate binding area of FgSULT1, superimposed with resveratrol (cyan sticks) and PAPS (gold sticks) from SULT1B1 (3CKL). Conserved active site residues H101 and K99 are shown as red sticks. Loops 1a, 1b, 2, and 3 are color-coordinated in panels A to C.

Although only distantly related to the enzymes in the animal and plant clades, FgSULT1 shares conserved motifs with the well-characterized SULT1 family members. Cytosolic SULTs all utilize the nucleotide donor PAPS as the sulfo group donor (3) and share three conserved motifs, the 5′-phosphosulfate-binding motif (PSB motif: TYPKSGT), the 3′-phosphate-binding motif (PB motif: YxxRNxxDxxVS) and the GxxGxxK/R motif that is crucial for the binding of both PAPS and the substrate (2, 18, 38, 49–51). All three of these motifs are also conserved in FgSULT1, with the PB motif showing some alterations (FxxRDxxDxxwS in FgSULT1, alterations underscored; Fig. 4A).

Most animal cytosolic SULTs exist as homodimers, while their plant counterparts are monomers (2, 3). The subunits of the homodimers in the animal cytosolic SULTs interact through the KTVE motif (KxxxTVxxxE), mutations of which convert the homodimer into a monomer (2, 52). This motif is not conserved in FgSULT1 (Fig. 4A), suggesting that this enzyme is monomeric. This prediction was confirmed by nondenaturing polyacrylamide gel electrophoresis and matrix-assisted laser desorption/ionization–time of flight mass spectrometry (MALDI-TOF MS) measurements of the mass of the purified enzyme, both of which were consistent with the calculated size (37.8 kDa) of the monomer (Fig. S6 in reference 36).

A homology structure model of FgSULT1 was constructed using the most similar protein in the Protein Data Bank (PDB) as the scaffold. This was the human cytosolic sulfotransferase SULT1B1 (PDB: 3CKL, 22% identity with FgSULT1 over 92% coverage), complexed with PAP or PAPS and the corresponding substrate, resveratrol (49). The homology structure model (model quality confidence score [C-score] of 0.22, estimated template modeling [TM] score with 3CKL of 0.74 ± 0.11, estimated root mean square deviation [RMSD] from 3CKL of 5.8 ± 3.6 Å) was further optimized using a molecular dynamic simulation. The resulting optimized model was superimposed on the experimentally determined structures of the human cytosolic sulfotransferase SULT1B1 (PDB: 3CKL; Fig. 4B), the Arabidopsis thaliana AtSOT18 (PDB: 5MEK; Fig. S7A in reference 36), which was solved in the presence of the cosubstrate PAPS and the substrate sinigrin (50), and StaL (51), the glycopeptide antibiotic SULT from Streptomyces toyocaensis (PDB: 4EEC; Fig. S7B in reference 36). Consistent with other SULT enzymes, FgSULT1 is predicted to feature a central four-stranded parallel *β*-sheet surrounded by 14 *α*-helices and two additional smaller *β*-strands (1, 38, 49–51) (Fig. 4B; Fig. S7 and S8 in reference 36). The PAPS binding site and the binding pose and conformation of this cosubstrate are all highly similar in the members of the cytosolic SULT superfamily. In contrast, there are substantial variations in the flexible loop regions in the proximity of the substrate (FgSULT1: loops 1b, 2, and 3; Fig. 4). Loop 1a is present (with low primary sequence similarities) in animal and plant SULTs (50, 53), but the corresponding amino acids are displaced by loop 1b from the proximity of the substrate in FgSULT1. Loop 1b appears to be unique to FgSULT1 and its homologues while absent from other SULTs (Fig. 4). Site-directed mutagenesis to introduce the T135A, the T137A, or the T139A mutations in loop 1b abolished FgSULT1 enzymatic activity (Fig. S9 in reference 36). This indicates the importance of this region, presumably for substrate access to the active site cavity. Although the primary amino acid sequences of loops 2 and 3 differ substantially among different SULTs, these regions were shown in both animal and plant enzymes to gate the substrate binding site and influence substrate specificity by restricting access to large substrates in the predominant “closed” conformation (49, 50). These loops are also present in FgSULT1 and may also regulate substrate promiscuity and regioselectivity. The predicted active site architecture of FgSULT1 suggests a relatively unrestricted access to the predicted catalytic residues and the cosubstrate PAPS (Fig. 4C). The volume of the predicted substrate binding cavity of FgSULT1 is 3,267 Å^3^ as measured by GHECOM 1.0 (with a maximum radius for the large probe set to 4 Å [Fig. S7C in reference 36]) (54). This cavity is much larger than those of the human cytosolic sulfotransferase SULT1B1 (992 Å^3^), the *S. toyocaensis* glycopeptide antibiotic SULT StaL (1,096 Å^3^), and the *A. thaliana* AtSOT18 (1,471 Å^3^). This large cavity in FgSULT1 is sufficient to allow the binding of a variety of phenolic xenobiotics and natural products, such as the various BDL congeners (Fig. S7C in reference 36). At the same time, this spacious cavity may disfavor the binding of the small, simple phenols and the “universal” SULT substrates 7-hydroxycoumarin 42 and *p-*nitrophenol 43 (Fig. S3 in reference 36).

The universally conserved active site residue H101 contacts both PAPS and the substrate: this residue was proposed to act as a catalytic base that deprotonates the phenolic hydroxyl group of the substrate (Fig. 4A) (55). Accordingly, the H101A mutant of FgSULT1 was found to be inactive. In animal SULTs, a conserved lysine (K99 in FgSULT1) also forms a hydrogen bond with the phenolic substrate and stabilizes the transient ternary intermediate (56). Although K99 is not essential for catalysis in the mouse SULT1A1, this residue may still facilitate the selection of phenolic substrates over other alcohols (1). Site-directed mutagenesis to generate the K99A mutation abolished the activity of FgSULT1, suggesting that this residue plays an essential role in substrate binding and/or sulfation catalysis in the case of this fungal enzyme (Fig. S9 in reference 36).

### Cytotoxicity evaluation of lasilarin and lasilarin 7-*O-sulfate*.

BDLs such as desmethyl-lasiodiplodin 5 show potent cytotoxicity against various cancer cells (57, 58). Thus, we evaluated the toxicities of lasilarin 1 and its 7-*O*-sulfate 1a against untransformed Vero cells (African green monkey kidney epithelium) and human cancer cell lines HeLa (cervical cancer), HepG2 (hepatocellular carcinoma), MCF-7 (breast cancer), and A549 (lung cancer) as the targets. However, no cytotoxicity was observed with either compound, even at the highest dose (50 μM).

## DISCUSSION

Research on sulfotransferases (SULTs) has focused on the twin roles that these enzymes play in phase II detoxification of various xenobiotics and the modulation of the activities of various endobiotics such as hormones, peptides, lipids, and carbohydrates. Naturally, most attention was paid to human SULTs, although an increasing number of enzymes from other mammals (especially rodents), insects, plants, and prokaryotes are also being analyzed (1–3, 50, 51). Although sulfation of a variety of small molecules by fungal cells is well known (12, 22) and a large number of hypothetical fungal proteins with predicted sulfotransferase domains have been deposited to data banks as a result of genome sequencing, to the best of our knowledge, no fungal SULT gene has ever been experimentally linked to any biotransformation reaction and no functionally validated fungal SULT enzyme has ever been deposited to the UniProtKB or other data bases. Thus, mining SULTs from filamentous fungi and comparing the structure-function relationships of these enzymes with other SULTs remains an unexplored topic. This stands in stark contrast with the widespread utilization of fungi and their characterized enzymes for biocatalysis in the biotech industry and the indispensable roles of these organisms in the detoxification of xenobiotics and the production of mycotoxins and other bioactive secondary metabolites in nature. Our work identifies, for the first time to the best of our knowledge, a PAPS-dependent phenolic SULT from the kingdom Fungi as the founding member of a new family within the cytosolic SULT superfamily. Phylogenetic analysis shows that members of this SULT family are distinct from other hypothetical fungal SULTs and predominantly appear in the *Fusarium* genus. Additional family members are present in selected species from the Hypocreales, Xylariales, and Microascales among the Sordariomycetes and in only two genera among the Dothideomycetes. Intriguingly, the family to which FgSULT1 belongs contains many predicted bacterial SULTs, mainly from Proteobacteria. Together with the patchy distribution of these enzymes in fungi and the lack of synteny in the fungal genomic loci harboring the genes for the FgSULT1 homologues, this may indicate horizontal gene transfer events from bacteria followed by limited retention of the transferred gene among selected fungal lineages. However, the presence of an intron in *fgsult1* and the absence of codon usage divergence in this gene compared to other F. graminearum genes suggests that any transfer event may have been ancient. While more extensive phylogenetic analyses may help to explain the complex evolutionary paths of these interesting genes in more detail, sequence comparison, structure modeling, and site-directed mutagenesis of selected residues reveals that FgSULT1 shares the classical fold of cytosolic SULTs, including the conserved catalytic residues, the PAPS-binding regions, and the flexible loops surrounding the substrate binding site (Fig. 4). Just like plant SULTs but different from the mammalian ones, FgSULT1 lacks the dimerization motif and retains a monomeric state. Taken together, structural and phylogenetic comparison of FgSULT1 with the human, plant, and prokaryotic SULTs highlights the remarkable evolutionary conservation of the architecture and catalytic mechanism of the distantly related proteins in the ancient cytosolic SULT superfamily.

We established that FgSULT1 from F. graminearum PH-1 is proficient in conjugating BDL congeners and other phenolic polyketides to yield the corresponding sulfate esters. Using 49 potential substrates with varied carbon skeletons, we detected sulfation with 14 scaffolds and isolated and elucidated the structure of one representative sulfated derivative, the novel compound lasilarin 7-*O*-sulfate 1a. Except for a 2,4-dihydroxybenzaldehyde motif that must be part of a more complex scaffold, no other, obvious structural constraint was evident for substrate acceptance by FgSULT1. Even with this motif present, the enzyme still shows idiosyncratic preferences for accepting or refusing closely related BDL analogues as potential substrates (cf. DHZ 3 and zearalenol 18). Considering the numerous homologues of FgSULT1 in fusaria that belong to the same subfamily of SULTs, suitable enzymes to sulfate other BDLs or related polyketides not recognized by FgSULT1 are likely to be discovered later.

We demonstrated that the generation of sulfated polyketides is feasible in a one-step format without tedious and environmentally problematic protection/deprotection chemistries by feeding preformed substrates to an S. cerevisiae biocatalyst expressing FgSULT1, or in a one-pot total biosynthetic format (59, 60) by coexpressing FgSULT1 with the BDL-producing PKS pairs in the same yeast chassis. Coupling the biosynthesis of polyketide scaffolds with the orthogonal tailoring provided by FgSULT1 expands the structural diversity of these compounds toward novel, unnatural natural products (uNPs). *De novo* production of BDL sulfates by microorganisms has not been reported earlier, with the notable exception of that for the fungal mycotoxin zearalenone. This BDL sulfoconjugate was found in the cultures of a zearalenone producer *Fusarium* strain when grown on rice medium (61). Considering that zearalenone 49 is not accepted as a substrate by FgSULT1, it is possible that the homologous SULT enzyme in the zearalenone producer strain has a different substrate range. Alternatively, the SULT activity may have been supplied by a different enzyme from the fungus or the plant seeds. Preformed zearalenone 49 can also be biotransformed to its sulfate ester by selected fungi from the genera *Rhizopus*, *Aspergillus*, and *Trichoderma* (6, 22); nevertheless, FgSULT1 shows negligible similarities to enzymes in the predicted proteomes of these fungi. Since homologues of the *fgsult1* gene reside in nonsyntenic regions in the genomes of *Fusarium*, *Xylaria*, *Hortea*, *Michrodochium*, and *Aureobasidium* spp., and in some cases may cluster with genes encoding enzymes in sulfur recycling, it is likely that the native function of FgSULT1 is not to be found in the biosynthesis of secondary metabolites. It is conceivable that these enzymes may be involved in the catabolism or phase II detoxification of potentially toxic metabolites (such as BDLs) encountered by these fungi in their native environments.

Importantly, BDL sulfate congeners and other sulfated uNP polyketides detected in this study display improved solubility and achieve more drug-like ClogP values (Table S3 in reference 36). Although sulfation often reduces bioactivity, some sulfoconjugates gain activity compared to their parent compounds or may show increased bioavailability and tissue distribution as prodrugs (4, 12, 18). Thus, such uNPs may be utilized for drug discovery in human and animal medicine and crop protection. Meanwhile, the synthetic biological methods exemplified here may also be utilized to generate inexpensive and accessible standards for the food industries and for environmental monitoring. Such standards may be valuable to detect masked mycotoxins (environmental toxins inactivated during phase II detoxification that are nevertheless easily reconverted to their active forms *in vivo*) or to expose precarcinogenic xenobiotics that are accidentally activated and rendered toxic by sulfation during human and animal metabolism or upon catabolism/detoxification by the microbiomes resident in animals or the environment.

## MATERIALS AND METHODS

### Strains, culture conditions, and chemical characterization of sulfate esters.

Escherichia coli DH10B and plasmid pJET1.2 (Thermo Fisher) were used for routine cloning and sequencing. Saccharomyces cerevisiae BJ5464-NpgA (*MATα ura3-52 his3-Δ200 leu2-Δ1 trp1 pep4::HIS3 prb1 Δ1.6R can1 GAL*) was used as the host for expression vectors based on plasmids YEpADH2p-URA, YEpADH2p-TRP, and YEpADH2p-LEU (23, 24, 26, 32, 41). Cultivation of Fusarium graminearum PH-1 and recombinant S. cerevisiae BJ5464-NpgA strains, primers used in this study, and details of the construction of expression vectors are described in references 23, 24, 26, 32, 36, and 41. Polyketide production was analyzed in three to five independent S. cerevisiae transformants for each recombinant yeast strain, and fermentations with representative isolates were repeated at least three times. Preparation of fermentation extracts, analysis by liquid chromatography-mass spectrometry (LC-MS), and product isolation were conducted as described in references 32 and 36. Sulfate ester products were characterized using HPLC-HRESIMS/MS and ^1^H and ^13^C NMR as described in reference 36.

### Protein structure modeling, phylogenetic analysis, and sequence comparison.

The FgSULT1 homology protein structure model was built with I-TASSER (62), and the resulting model was optimized using Discovery Studio v2.7 as described in reference 36. Protein structures were compared using the Dali server (63), cavity volumes were measured using the GHECOM 1.0 server (54), and sequence alignments were performed with MUSCLE v3.6 (64). Phylogenetic relationships were reconstructed using the maximum likelihood and the neighbor joining methods in MEGA v5.2 (65). Statistical support was generated by bootstrap analysis with 1,000 pseudoreplicates. SULTs with confirmed functions were obtained from the UniProtKB database (Table S6 in reference 36).

### Protein purification, oligomer status, and enzyme activity assays.

The His-tagged FgSULT1 protein was purified to homogeneity (as judged by SDS-PAGE) using Ni^2+^-NTA column affinity chromatography. Native PAGE and MALDI-TOF were used to measure the molecular mass of FgSULT1. Enzyme activity assays were performed at 30°C in 100-μl reaction mixtures containing 50 mM Tris-HCl (pH 8.0), 10 mM MgCl_2_, 0.2% Tween 20, 1 mM lasilarin 1 as the substrate, 1 mM PAPS as the sulfate donor cosubstrate, and 10 μg FgSULT1. Reactions were stopped by extraction with ethyl acetate.
